# Immune biomarker-based enrichment in sepsis trials

**DOI:** 10.1186/s13054-020-2774-1

**Published:** 2020-02-19

**Authors:** Thibaud Spinetti, Christian Meisel, Stephan von Gunten, Joerg C. Schefold

**Affiliations:** 1 Department of Intensive Care Medicine, Inselspital, Bern University Hospital, University of Bern, Bern, Switzerland; 2 Department of Medical Immunology, Charité University Medicine Berlin, Berlin, Germany; 3 Institute of Pharmacology, University of Bern, Bern, Switzerland

Dear Editor

We read with great interest the study by Anderson et al. recently published in *Critical Care* [1]. The study assessed whether soluble mediators (IL-8, sTNFR1, and Ang-2) could be used as biomarkers to “enrich” subject populations with higher mortality risk in subsequent clinical trials. The authors addressed “immunocompetence” of patients using clinical parameters (APACHE-II score and/or presence of ARDS). They found that both IL-8 and sTNFR1 (but not Ang-2) were suitable for identification of patients with higher mortality risk and concluded that IL-8 and sTNFR1 can be used as “prognostic enrichment factors” in future clinical sepsis studies.

Biomarker-based prognostic enrichment appears important to select sample populations with a greater likelihood of having improved clinical outcomes following a given therapeutic intervention. Although sTNFR1 and IL8 levels may be associated with higher mortality in certain sepsis patients, however, selection of the correct “enrichment markers” should be performed cautiously and based on a solid underlying biological rationale. This may, for example, be of particular importance in the field of clinical sepsis trials testing immunomodulatory interventions where assessment of the pleiotropic cytokine IL-8 would likely introduce considerable bias and should thus not be used to stratify respective patient populations. Failure of an adequate peri-interventional characterization may at least partly explain the failure of a number of previous sepsis trials testing immunological interventions (e.g., corticosteroids, strategies testing anti-TNF or anti-LPS). In the study by Anderson et al., “immunocompetency” in sepsis patients was defined using clinical criteria. However, it seems that immunocompetency, i.e., (functional) immune phenotype, cannot be assessed by predominantly clinical parameters and should be based on comprehensive functional immune markers (e.g., mHLA-DR expression [2–4]) in order to identify individuals who would benefit most from a given intervention.

We are well aware that the focus of the article was to address the important question of whether biomarker-based enrichment would lead to better stratification of future trial cohorts.

While we appreciate the insights provided by Anderson et al., we believe that it will be crucial (and challenging) to continue the quest for the “correct” enrichment markers to succeed in the design of novel therapeutic interventions, which may require more extensive reverse translational research and personalized treatment approaches [5]. In the light of failure of a large number of previous clinical sepsis trials, it seems apparent that biomarker enrichment using appropriate mediators is needed and may open several avenues towards more personalized treatment approaches in sepsis.

## Data Availability

Not applicable.

## Abbreviations

IL-8: Interleukin 8
sTNFR1: Soluble tumor necrosis factor receptor-1
Ang-2: Angiopoietin-2
APACHE: Acute Physiology, Age, Chronic Health Evaluation
ARDS: Acute Respiratory Distress Syndrome
TNF: Tumor necrosis factor
LPS: Lipopolysaccharide
